# Multifunctional Silk Fibroin–Curcuminoid Films Combining Regenerative and Antioxidant Properties with pH Sensing for Wound Dressing Applications

**DOI:** 10.3390/biomimetics11090635

**Published:** 2026-09-05

**Authors:** Rebecca Pellegrino, Maria Rosa Iaquinta, Annalia Masi, Mauro Pollini, Federica Paladini

**Affiliations:** 1 Department of Engineering for Innovation, University of Salento, 73100 Lecce, Italy; rebecca.pellegrino@unisalento.it (R.P.); annalia.masi@unisalento.it (A.M.); 2 Department of Experimental Medicine, University of Salento, 73100 Lecce, Italy; mariarosa.iaquinta@unisalento.it

**Keywords:** smart wound dressing, silk fibroin, curcuminoids, pH changes, wound healing

## Abstract

The management of chronic wounds represents one of the major challenges in regenerative medicine, as the healing process can be compromised by infections, oxidative stress, and persistent inflammation. In this context, wound pH serves as an important biomarker of tissue status, highlighting the need for smart dressings capable of promoting regeneration while simultaneously monitoring the wound microenvironment. In this study, biomimetic silk fibroin films functionalized with curcuminoids extracted from *Curcuma longa* were developed and characterized through spectroscopic, swelling/degradation, antioxidant, colorimetric, and biological assays, with the aim of obtaining a multifunctional dressing with regenerative properties and pH responsiveness. The results showed that curcuminoids were physically incorporated into the protein matrix without altering its chemical structure. The films exhibited a high absorption ability and antioxidant activity in the initial stages, and a clear and reversible color change in response to pH. Biological assays on 3T3 fibroblasts further confirmed the high cytocompatibility of the materials and their ability to support cell migration and wound closure in vitro. The developed films represent a promising biomimetic platform for advanced wound dressings, capable of combining support for tissue regeneration, antioxidant protection, and visual monitoring of wound status through the detection of pH changes.

## 1. Introduction

The advanced management of chronic and acute wounds is a significant challenge in regenerative medicine and biomaterial bioengineering due to the complexity of the tissue microenvironment involved in the healing process [1,2,3]. Wound healing is a dynamic, multi-phase phenomenon governed by the intricate interplay between cells, the extracellular matrix (ECM), biochemical mediators, and local physicochemical parameters, including pH [4]. Under physiological conditions, wound healing proceeds through the coordinated phases of hemostasis, inflammation, proliferation, and remodeling [5,6]. However, alterations to the wound microenvironment, such as bacterial infection, oxidative stress, and persistent inflammation, can impair the regenerative process [7,8,9]. In this context, pH emerges as a key biomarker of wound status: shifts towards more alkaline values are often linked to infection, delayed healing, and chronicity, whereas a mildly acidic environment encourages re-epithelialization and angiogenesis and controls microbial proliferation [10,11,12]. In recent years, the design of “smart” or biofunctional dressings has attracted growing interest as a strategy to combine therapeutic support with monitoring of the wound microenvironment [13,14,15,16]. Among the materials most widely studied in this field, silk fibroin stands out due to its excellent biological and structural properties. Derived primarily from *Bombyx mori*, fibroin is a natural protein that is biocompatible and biodegradable, while also possessing favorable mechanical and processability characteristics [17,18,19,20]. Furthermore, its ability to form membranes, hydrogels, scaffolds, and thin films makes it particularly suitable for developing biomimetic systems for wound healing applications [21,22,23,24]. From a biomimetic perspective, fibroin films can replicate certain ECM functions, providing a substrate that promotes cell adhesion, maintains a moist environment, and protects the injured site [25,26].

Incorporating bioactive molecules into the fibroin matrix represents a further step towards developing multifunctional devices [27]. Curcuminoids, and in particular curcumin, have attracted significant interest due to their antioxidant, anti-inflammatory, antimicrobial, and pro-regenerative properties [28]. This natural polyphenol, extracted from the *Curcuma longa* rhizome, can modulate various molecular pathways involved in the inflammatory response and tissue repair [29,30]. Despite their limitations regarding bioavailability and stability in aqueous environments, incorporating curcuminoids into protein-based polymer matrices such as fibroin can improve its protection, controlled release, and local efficacy, making it a promising candidate for advanced wound dressings [31,32,33].

In addition to its therapeutic role, curcuminoids possess a particularly interesting characteristic for the development of “responsive” materials: their chemical structure is sensitive to pH variations, which can alter the molecule’s optical and chromatic properties, appearing yellow at a pH between 1 and 7 and reddish orange at a pH higher than 8 [34,35]. This behavior means that curcuminoids can be used not only as a bioactive agent but also as a visual or spectroscopic indicator of the status of the wound microenvironment [36,37]. Combining fibroin with curcuminoids enables the design of biomimetic films that can perform a dual function: promoting wound healing through protective and therapeutic action, while simultaneously signaling local pH changes [38,39,40]. This offers a potential tool for the early monitoring of pathological conditions such as infection or persistent inflammation. Research into these biomaterials thus falls within an interdisciplinary field combining biomimetics, materials science, and regenerative medicine.

In this context, the present study was designed to develop and characterize silk fibroin-based films functionalized with naturally extracted curcuminoids, with the aim of combining tissue-supporting properties with an optical response to pH changes in the wound microenvironment. The study was structured to investigate the effects of curcuminoid incorporation on the physicochemical, functional and biological properties of the fibroin matrix in the form of thin films for wound healing applications.

## 2. Materials and Methods

### 2.1. Materials

The aqueous silk fibroin solution (5% *w*/*v*) from *Bombyx mori* cocoons was kindly provided by Caresilk S.r.l.s. (Lecce, Italy). Methanol (≥99.9%, MW 32.04) and phosphate-buffered saline (PBS) were purchased from Sigma-Aldrich (Saint-Louis, MO, USA); absolute ethanol was purchased from ITW Reagents S.r.l. (Milan, Italy); and turmeric powder was supplied from a local supermarket.

All aqueous solutions were prepared using distilled water.

### 2.2. Curcuminoids Extraction Method

Curcuminoids from turmeric root (*Curcuma longa*) were extracted using methanol as the extraction solvent. The turmeric powder was weighed out and suspended in methanol at a 1:10 (*w*/*v*) ratio. The mixture was stirred overnight (ON) and covered with aluminum foil, as the compounds of interest are sensitive to light. The resulting suspension was then filtered, and the filtrate was dried on a glass surface. The dried extract was collected as a powder and used for further tests (CUR). The yield of the extraction process (I) was expressed as the percentage of the theoretical curcuminoid content recovered in the extract and calculated according to Equation (1):

(1) $$I\;(\%)=\frac{w_{d}}{w_{i}\times 0.04}\times 100,$$

where *w_i_* is the initial weight of the *Curcuma longa* powder, *w_d_* is the weight of the dried extract obtained after solvent evaporation, and 0.04 represents the average of curcuminoid content (4% *w*/*w*) of turmeric powder, as reported in the literature [41].

### 2.3. Silk Fibroin–Curcuminoid Film Preparation

Thin, non-porous films were prepared by air drying. The curcuminoid powder obtained from the extraction process was suspended in methanol at a concentration of 10 mg/mL. Then, 1 mL of the resulting curcuminoid suspension was incorporated into 10 mL of a silk fibroin aqueous solution (5% *w*/*v*), resulting in a final concentration of curcuminoids of approximately 1 mg/mL. The mixture was stirred for 4 h to promote homogeneous dispersion of curcuminoids within the silk fibroin solution and subsequently transferred into polystyrene dishes (10 cm diameter) and allowed to dry in a laminar flow hood for 72 h at room temperature (RT), obtaining non-porous silk fibroin-based films containing curcuminoids. The obtained films were crystallized by immersion in 20 mL of methanol for 15 min. The resulting samples were labeled as SF_CUR/2MetOH. The methanol solution recovered after the crystallization process was analyzed to quantify the amount of curcuminoids released during the treatment. The recovered solution was measured for absorbance at 420 nm using a Multimode Plate Reader EnVision (PerkinElmer, Waltham, MA, USA). The curcuminoid concentration was determined for eight samples using a previously generated calibration curve and reported as the mean ± Standard Deviation (S.D.). Control samples were also prepared by adding 1 mL of pure methanol to the silk fibroin solution instead of the curcuminoid suspension and crystallizing the obtained films for 15 min in methanol (labeled as SF/2MetOH). To investigate the effect of the addition of methanol in the solution and the subsequent crystallization treatment, additional samples were prepared and analyzed: (i) untreated silk fibroin films (SF), (ii) silk fibroin films containing methanol without subsequent crystallization (SF/MetOH), and (iii) silk fibroin–curcuminoid films without subsequent crystallization (SF_CUR/MetOH).

### 2.4. Films Characterization

#### 2.4.1. Fourier-Transform Infrared Spectroscopy (FT-IR)

FT-IR spectra were recorded for all samples (CUR, SF, SF/MetOH, SF/2MetOH, SF_CUR/MetOH, SF_CUR/2MetOH) to evaluate the effects of methanol-induced crystallization and curcuminoid incorporation on the chemical structure of the silk fibroin films. Measurements were performed in Attenuated Total Reflectance (ATR) mode using a Jasco FT/IR-6300 spectrometer (Thermo Fisher Scientific, Waltham, MA, USA) at a wavelength range of 500–4000 cm^−1^ with a resolution of 4 cm^−1^. FT-IR data were analyzed using OriginPro software (OriginLab, version 8, Northampton, MA, USA).

#### 2.4.2. Thermal Analysis

Differential scanning calorimetry (DSC) was performed on all samples (CUR, SF, SF/MetOH, SF/2MetOH, SF_CUR/MetOH, SF_CUR/2MetOH) using a Q2000 DSC instrument (TA Instruments, New Castle, DE, USA). Approximately 5 mg of each sample were weighed into hermetically sealed aluminum crucibles and heated from 25 °C to 400 °C under an inert nitrogen atmosphere at a flow rate of 50 mL min^−1^ and a heating rate of 5 °C min^−1^. An empty aluminum pan was used as the reference. DSC data were analyzed using OriginPro software (OriginLab, version 8, Northampton, MA, USA).

#### 2.4.3. Swelling

To investigate the ability of films to absorb liquids, all samples (SF, SF/MetOH, SF/2MetOH, SF_CUR/MetOH, SF_CUR/2MetOH) were cut into rectangular specimens, measuring 10 mm wide by 20 mm long, weighed in the dry state, and immersed in 0.01 M PBS at RT. At predetermined time points (30 min, 1 h, 2 h, 3 h, 5 h, and 24 h), they were removed and immediately weighed again in the swollen state. The degree of swelling, or water uptake, was gravimetrically calculated in terms of swelling ratio using Equation (2):

(2) $$Swelling\;degree\;(\%)=\frac{w_{w}-w_{d}}{w_{d}}\times 100,$$

where *w_w_* is the weight of the swollen sample and *w_d_* is the initial dry weight. The experiments were performed in triplicate, and results were reported as the mean ± S.D.

#### 2.4.4. Evaluation of In Vitro Degradation and Curcuminoids Release

The stability in aqueous solution of the selected films (SF/2MetOH and SF_CUR/2MetOH) was investigated under physiologically relevant conditions. Rectangular film samples (10 mm × 20 mm) were weighed in the dry state and incubated in 10 mL of 0.01 M PBS at 37 °C. At predetermined time intervals, the films’ resistance to degradation was quantified using a colorimetric assay. In brief, aliquots of the incubation solution were taken after 1, 3 and 7 days to determine the concentration of solubilized silk fibroin using the bicinchoninic acid (BCA) colorimetric assay (QuantiPro^TM^ BCA kit, Sigma-Aldrich, Saint Louis, MO, USA). Absorbance was measured at 562 nm using a Multimode Plate Reader EnVision (PerkinElmer, Waltham, MA, USA). Other aliquots were also recovered and centrifuged at 13,000 rpm. The pellet was recovered and resuspended in methanol to evaluate the release of curcuminoids by measuring the absorbance at 420 nm using a Multimode Plate Reader EnVision (PerkinElmer, Waltham, MA, USA). The curcuminoid concentration was determined for three samples using a previously generated calibration curve and reported as the mean ± S.D.

#### 2.4.5. Scavenging Activity of Silk Fibroin–Curcuminoid Films Using DPPH

SF_CUR/2MetOH samples and SF/2MetOH controls were cut into small pieces (approximately 5 mg each) and immersed in 1 mL of 0.01 M PBS at 37 °C into 2 mL Eppendorf tubes. At predetermined time points (1, 3, and 7 days), the buffer was completely removed, and 1 mL of 2,2-diphenyl-1-picrylhydrazyl (DPPH) radical solution (3.95 mg dissolved in 100 mL methanol) was added to start the antioxidation test. After 60 min of incubation at RT, the films were removed and the absorbance of the DPPH solution was measured at 517 nm using a Multimode Plate Reader EnVision (PerkinElmer, Waltham, MA, USA). The absorbance value was compared with the absorbance of the DPPH solution incubated in the presence of control films to obtain the percentage scavenging value according to Equation (3):

(3) $$DPPH\;scavenging\;activity\;(\%)=\frac{A_{0}-A_{s}}{A_{0}}\times 100,$$

where *A*_0_ is the absorbance of DPPH solution in the presence of control films and *A_s_* is the absorbance of DPPH solution in the presence of films containing curcuminoids.

#### 2.4.6. pH Responsiveness of Silk Fibroin–Curcuminoid Films

The pH responsiveness of the silk fibroin–curcuminoid films was studied by taking small patches of the SF_CUR/2MetOH films (diameter ~1 cm) and introducing them in three different pH buffer solutions in order to simulate the different stages of the wound healing process: (i) PBS (pH 7.4), (ii) an acidic buffer (pH 5.5), prepared using 0.105 g of citric acid monohydrate and 0.390 g of trisodium citrate dihydrate dissolved in 40 mL of distilled water (Sigma Aldrich; Saint-Louis, MO, USA), and (iii) an alkaline buffer (pH 8.5), prepared using 0.242 of Tris base and 200 μL of 1 M acid citric in 40 mL of distilled water (Sigma Aldrich; Saint-Louis, MO, USA). The color change in films was quantitatively evaluated using the Commission International de l’Eclairage (CIE) L* a* b* system in ImageJ, where L* corresponds to lightness (ranging from 0 [black] to 100 [white]), a* corresponds to the red-green axis (from positive to negative values, respectively), and b* corresponds to the yellow–blue axis (from positive to negative values, respectively) [42]. Three identical regions of interest (ROIs) were selected in different areas of each film, and the mean ± S.D. L*, a*, b* values were calculated. The color variation between neutral pH and alkaline and acidic conditions was determined using Equation (4):

(4) $$∆E=\;\sqrt{{\left(∆L\right)}^{2}+{\left(∆a\right)}^{2}+{\left(∆b\right)}^{2}},$$

### 2.5. Biocompatibility Evaluation of Silk Fibroin–Curcuminoid Films

The biocompatibility of silk fibroin–curcuminoid films was evaluated using 3T3 fibroblasts. Specifically, the films were tested maintaining 3T3 fibroblasts on the most performant samples: SF/2MetOH (used as a control) and SF_CUR/2MetOH films. Before biological evaluation, SF/2MetOH and SF_CUR/2MetOH films were sterilized by two consecutive 20 min ethanol treatments, followed by three washes with PBS for 10 min each, and subsequently incubated in cell culture medium for 30 min before the cell-based assays. Tissue culture polystyrene (TCPS) was included as control. 3T3 fibroblasts were expanded in Dulbecco’s Modified Eagle Medium (DMEM; Sigma Aldrich) supplemented with 10% fetal bovine serum (FBS) and 1% antibiotic solution (100 U/mL penicillin, 100 μg/mL streptomycin). Cells were maintained in a humidified incubator (Heracell, Thermo Scientific, Waltham, MA, USA) at 37° C in an atmosphere of 5% CO_2_. The culture medium was replaced every three days. After expansion, 3T3 fibroblasts were seeded in 24 well plates (15 × 10^4^ cells/well). For each experimental group, three independent replicates were performed.

#### 2.5.1. MTT Assay

3T3 fibroblast metabolic activity was quantified through the MTT assay [3-(4,5-dimethylthiazol-2-yl)-2,5-diphenyltetrazolium bromide; Sigma Aldrich] as reported [43]. Cells were cultured in a 24-well plate in contact with the SF/2MetOH and SF_CUR/2MetOH films, while TCPS was used as the control. At each time point of analysis, a stock MTT solution (5 mg/mL in PBS) was diluted in culture medium in order to obtain a final concentration of 0.5 mg/mL. Following the incubation (4 h, 37 °C), the formazan deposits were solubilized with Dimethyl Sulfoxide (DMSO). The optical density was measured at 540 nm using a V-1200 spectrophotometer (Avantor, Inc., VWR, Radnor Township, PA, USA). The metabolic activity of 3T3 fibroblasts was quantified after 3, 5, and 7 days of culture.

#### 2.5.2. Cytoskeleton Architecture Analysis

The cytoskeletal structure of 3T3 fibroblasts cultured in contact with SF/2MetOH and SF_CUR/2MetOH films was evaluated at day 7. 3T3 fibroblasts were fixed with 4% paraformaldehyde for 20 min at RT. Cell membranes were subsequently permeabilized by treatment with 0.5% Triton X-100 for 10 min, followed by PBS rinsing. To visualize the actin filaments, 3T3 fibroblasts were stained with tetramethylrhodamine isothiocyanate (TRITC)-conjugated phalloidin (Sigma Aldrich) [44]. Cell nuclei were stained using 0.5 mg/mL 4′,6-diamidino-2-phenylindole (DAPI; Invitrogen, Carlsbad, CA, USA). The Axio Vert A1 microscope (Zeiss) was employed to collect fluorescence images, while image analysis was performed using AxioVision software (Zeiss ZEN 3.11).

#### 2.5.3. Live and Dead Staining Assay

The Live and Dead staining assay was further used to analyze cell viability [45]. Live/Dead assay was conducted on 3T3 fibroblasts cultured in contact with SF/2MetOH and SF_CUR/2MetOH films, and TCPS for 7 days. For staining, 3T3 fibroblasts cultured directly on glass coverslips were incubated with a staining solution containing 2 μmol/L calcein-AM (acetoxymethyl ester of calcein) and 2 μmol/L propidium iodide in PBS at 37 °C for 15 min in order to stain live and dead cells, respectively. Fluorescence images were subsequently acquired using a fluorescence microscope (Axio Vert A1, Zeiss, Oberkochen, Germany) at 20× magnification. Image visualization and analysis were performed using AxioVision software (Zeiss ZEN 3.11).

In addition to qualitative assessment of viability, quantitative morphological parameters were obtained from the fluorescence images of living cells. Individual 3T3 fibroblasts were manually outlined using ImageJ software (version 1.54c14; National Institutes of Health, Bethesda, MD, USA). The cell area, major axis length, perimeter, aspect ratio, and circularity were determined [46]. The aspect ratio was calculated as the ratio between the major and minor axes, while the circularity was calculated according to Equation (5):

(5) $$Circularity=\;4\pi \;\times \;\frac{Cell\;Area}{{Perimeter}^{2}}\;,$$

For the morphological analysis, sixty cells from three independent experiments were analyzed for each experimental condition. Cell number was determined manually.

#### 2.5.4. Wound-Healing Assay

The migratory behavior of 3T3 fibroblasts was investigated by a wound-healing assay. 3T3 fibroblasts were initially seeded in 24-well plates (15 × 10^4^ cells/well) and cultured at 37 °C with 5% CO_2_ until reaching 90% confluence. A linear scratch was realized using a 1000 μL pipette tip, followed by exposure of the cells to SF/2MetOH and SF_CUR/2MetOH films. Images were acquired at 10× magnification after scratch generation (T0) and after 2 and 3 days (T1 and T2, respectively) using a fluorescence microscope (Axio Vert A1, Zeiss, Oberkochen, Germany). The images were acquired using AxioVision software (Zeiss ZEN 3.11). Images of cell migration were analyzed using ImageJ software [47,48].

### 2.6. Statistical Analysis

Results are expressed as the mean ± S.D. based on the indicated number of experiments. The statistical analysis was conducted by using One- and Two-way ANOVA. In all comparisons, *p* < 0.05 was considered statistically significant, and the *p*-values are reported when present. All ANOVA post hoc analyses were performed using Tukey’s test.

## 3. Results

### 3.1. Curcuminoids Extraction 

Curcuminoids were successfully recovered as dried powder after the extraction process. Based on literature research, *Curcuma longa* rhizome powder typically contains approximately 3–5% (*w*/*w*) total curcuminoids, with values of up to 10% depending on cultivar, geographical origin, and process conditions [41,49,50]. Assuming an average curcuminoid content of 4% (*w*/*w*), the theoretical recovery of curcuminoids obtained in the present extraction was calculated to be 88 ± 5%.

### 3.2. Silk Fibroin–Curcuminoid Film Preparation

**Figure 1:**
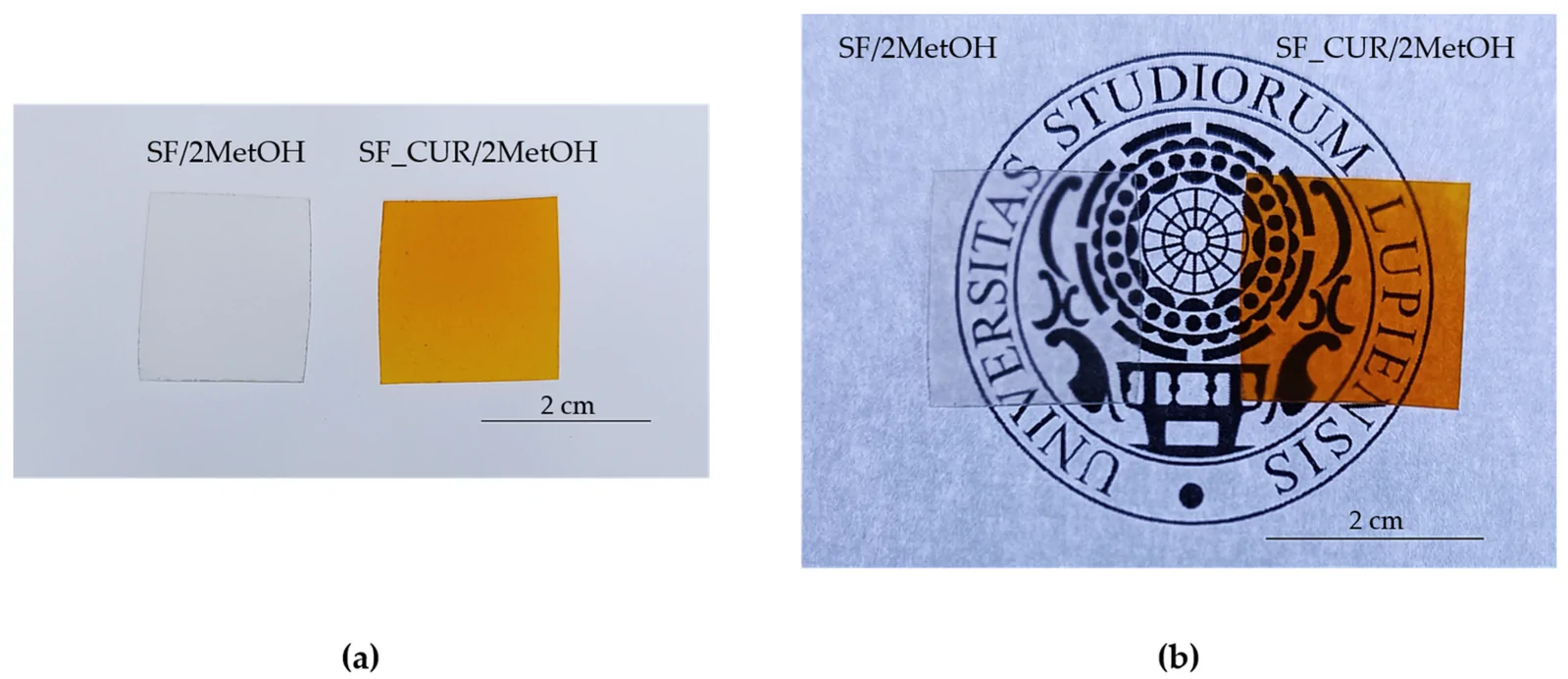
(**a**) Visual color difference between SF/2MetOH and SF_CUR/2MetOH and (**b**) transparency of SF/2MetOH and SF_CUR/2MetOH.

Silk fibroin–curcuminoid films were successfully obtained, and the presence of curcuminoids was initially confirmed by visually observing a different color of the SF_CUR/2MetOH film compared to the SF/2MetOH control film, from completely transparent to orange in the presence of curcuminoids, while maintaining their transparency, as observed in Figure 1.

The crystallization step of silk fibroin involved the immersion of films in methanol for 15 min. Since methanol is also a solvent for curcuminoids, a partial loss of curcuminoids during this step was expected. To quantify this loss, the methanol recovered after the crystallization of eight films was analyzed by UV-Vis spectroscopy. The absorbance was measured at 420 nm, corresponding to the maximum absorption wavelength of curcuminoids, and the concentration of curcuminoids was calculated using a calibration curve previously established (A = 0.0108 C + 0.0046, R^2^ = 0.9949, where A is the absorbance and C is the concentration of curcuminoids in µg/mL). Based on this calibration, the concentration of curcuminoids released into methanol during crystallization was estimated to be 2.66 ± 0.40 µg/mL.

### 3.3. Physicochemical and Functional Characterization of the Films

Silk fibroin–curcuminoid films were characterized before and after methanol-induced crystallization (SF_CUR/MetOH and SF_CUR/2MetOH, respectively) to evaluate whether the addition of methanol in the solution (1:10 *v*/*v*) was sufficient to obtain stable films suitable for biomedical applications. All results were compared with those obtained for the corresponding control samples without curcuminoids (SF/MetOH and SF/2MetOH) and the untreated silk fibroin films (SF).

**Figure 2:**
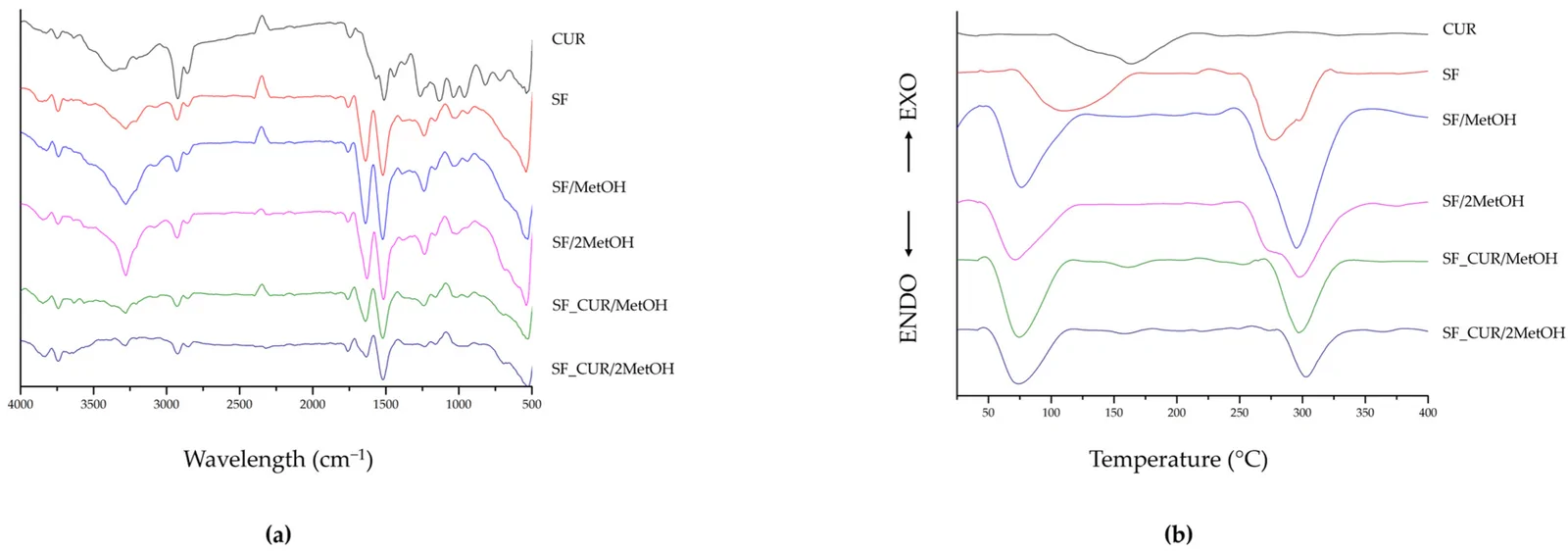
Films characterization: (**a**) FT-IR spectra of CUR, SF, SF/MetOH, SF/2MetOH, SF_CUR/MetOH and SF_CUR/2MetOH; (**b**) DSC curves of CUR, SF, SF/MetOH, SF/2MetOH, SF_CUR/MetOH and SF_CUR/2MetOH.

The FT-IR spectra of the extracted curcuminoids and the prepared films are shown in Figure 2a. In the present study, FT-IR analysis was used to investigate the extracted curcuminoid powder, to observe the effect of curcuminoid incorporation and methanol-induced crystallization on the produced films, and to verify possible interactions between the curcuminoids and the protein. The spectrum of the extracted curcuminoids (CUR) showed the presence of characteristic curcuminoid peaks. The main signals in the spectrum of curcuminoids were observed at 3367 (O-H stretching vibrations), 2924 and 2857 (-CH- asymmetric stretching), 1565 (C=C aromatic stretching), 1511 (Benzene ring bending vibration), 1442 and 1369 (CH bending), and 1133 (C-O stretching) cm^−1^, which match those previously described in the literature [51]. The untreated silk fibroin film (SF) presented the characteristic absorption peaks of Amide I, Amide II and Amide III at 1650 cm^−1^ (-CO- and -CN- stretching), 1531 cm^−1^ (-NH bending), and 1230 cm^−1^ (-CN- stretching), respectively, that confirmed a random coil and α-helix conformation (silk I structure) of the protein [52,53]. The addition of methanol into the solution (0.1 mL/mL), as performed in this study, did not alter the chemical structure of untreated silk fibroin, as demonstrated by the similar FT-IR profile observed for the SF/MetOH sample. Conversely, when immersed in methanol for 15 min, silk fibroin rearranged into a β-sheet structure (silk II), with a shift in characteristic peaks of the Amides I from 1650 to 1627 cm^−1^, the Amide II band from 1531 to 1515 cm^−1^, and the Amide III from 1230 to 1238 cm^−1^, due to increased intermolecular hydrogen bonding and molecular rearrangement within the fibroin matrix [24,54,55,56]. The same spectral changes were observed in the films containing curcuminoids, with the SF_CUR/2MetOH sample exhibiting the characteristic protein transition induced by methanol treatment with respect to SF_CUR/MetOH. However, intense absorbance of the protein signals, in addition to a relatively low curcuminoid-to-fibroin mass ratio, masked the curcuminoid signals in these films. Similar masking effects have been reported for other silk-fibroin-based composite systems [57]. The absence of new peaks or significant spectral shifts suggested that curcuminoid incorporation did not significantly alter the chemical structure of silk fibroin, indicating that the compounds were physically incorporated within the protein matrix rather than forming new chemical bonds.

Thermal analysis in Figure 2b confirmed the structural changes observed by FT-IR spectroscopy. With respect to untreated silk fibroin (SF), the addition of methanol into the fibroin solution (SF/MetOH) resulted in a slight shift in the main endothermic degradation peak toward higher temperatures. This effect became more evident after methanol-induced crystallization for 15 min, with the degradation peak shifting from 277 °C to 298 °C, indicating an improvement in the thermal stability of the fibroin matrix that can be attributed to the methanol-induced formation and increased organization of *β*-sheet structures, which results in a more ordered and thermally stable fibroin network. Similar behavior was observed for silk fibroin–curcuminoid films, where the degradation peak shifted from approximately 290 °C in SF_CUR/MetOH to 303 °C after the crystallization in methanol (SF_CUR/2MetOH). Compared to silk fibroin films without curcuminoids, both SF_CUR/MetOH and SF_CUR/2MetOH exhibited a further slight increase in the degradation temperature compared to untreated SF, suggesting a possible stabilizing effect of curcuminoids within the fibroin matrix related to the possible presence of weak physical interactions between them even if no new chemical interactions were detected by FT-IR analysis. Finally, the extracted curcuminoids exhibited a broad endothermic peak at approximately 165 °C, which is consistent with the characteristic thermal transition reported for curcuminoids [58,59]. The broad shape of this peak suggested a heterogeneous structure of the extracted compound. This transition was only weakly visible in the SF_CUR/MetOH and SF_CUR/2MetOH films, because of the relatively low curcuminoid content compared with the silk fibroin matrix. For all samples, the first endothermic peak at low temperatures was associated with the evaporation of absorbed water and residual moisture of films.

**Figure 3:**
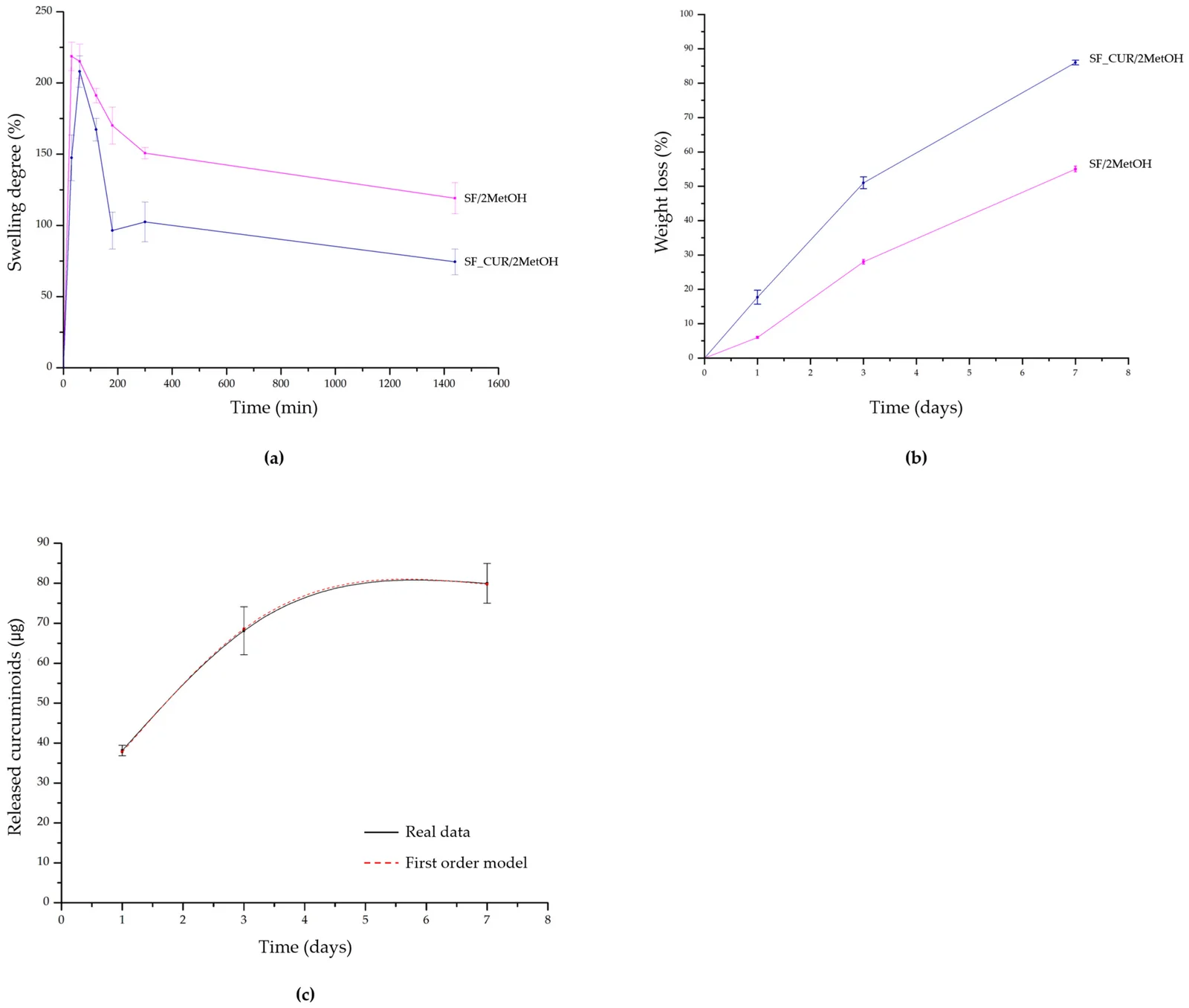
Films characterization: (**a**) Swelling degree of SF/2MetOH and SF_CUR/2MetOH; (**b**) Weight loss of SF/2MetOH and SF_CUR/2MetOH; (**c**) Released curcuminoids from SF_CUR/2MetOH.

To evaluate the behavior of the films under simulated physiological conditions, their capacity to absorb liquids and stability over time were investigated through swelling and degradation studies. The swelling test was performed for all samples (SF, SF/MetOH, SF_CUR/MetOH, SF/2MetOH, and SF_CUR/2MetOH) but the first three formulations dissolved immediately upon immersion in PBS, preventing swelling measurement at the selected time points. On the contrary, SF/2MetOH and SF_CUR/2MetOH maintained their structural integrity and exhibited a similar swelling profile (Figure 3a). Both samples showed a rapid increase in weight, reaching swelling values of approximately 218% at 30 min and 208% at 1 h, respectively. The time needed to reach the maximum was different: silk fibroin–curcuminoid samples took more time, probably due to the hydrophobic nature of curcuminoids. Subsequently, a gradual decrease in swelling was observed over time, which can be attributed to the progressive degradation of the silk fibroin matrix. The decrease was more evident for silk fibroin–curcuminoid samples, which reached a final swelling value of 74% compared with 119% for SF/2MetOH. This behavior may be related to the combined effects of fibroin degradation and the release of curcuminoids from the protein matrix during incubation. In fact, the degradation profile revealed a significantly higher protein loss for the curcuminoid-loaded films (SF_CUR/2MetOH) compared with the control films (SF/2MetOH) at all investigated time points (Figure 3b). Specifically, after 1 day of incubation, SF_CUR/2MetOH exhibited a degradation of approximately 17%, whereas SF/2MetOH showed only 6% degradation. The degradation of SF_CUR/2MetOH further increased to approximately 51% and 86% after 3 and 7 days, respectively, while the corresponding values for the control films reached 28% and 55%. Moreover, for SF_CUR/2MetOH films a high release of curcuminoids in PBS was observed in the first stages, calculated using a calibration curve previously established (A = 0.0108 C + 0.0046, R^2^ = 0.9949, where A is the absorbance and C is the concentration of curcuminoids in µg/mL), following a first-order kinetic model, described by the equation *C_t_* = *C*_∞_(1 − *e*^−^*^k^*_^1^_*^t^*). Specifically, the model parameters were *C*_∞_ = 80.71 and *k*_1_ = 0.6317, with an R^2^ value of 0.9997 indicating an excellent agreement between the experimental release profile and the model (Figure 3c). This behavior suggested that the release rate was dependent on the amount of curcuminoids remaining within the film matrix, resulting in a relatively faster release in the initial stages followed by a slower release as the amount of retained compound decreased [60].

**Figure 4:**
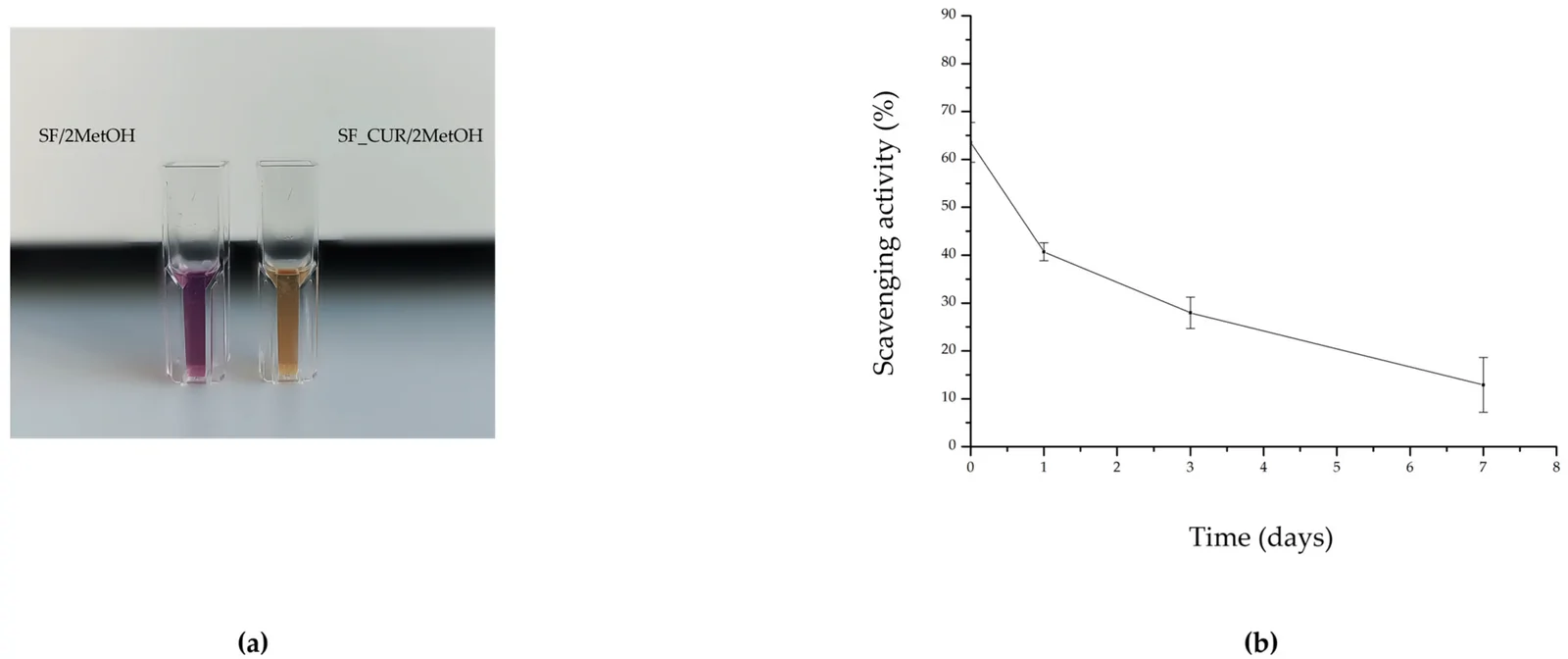
Films characterization: (**a**) example of a visual decolorization in DPPH assay; (**b**) scavenging activity of SF_CUR/2MetOH films.

The antioxidant activity of silk fibroin–curcuminoid films was examined using a DPPH assay. In this method, antioxidant molecules donate hydrogen atoms to the stable DPPH radical, leading to the formation of the reduced non-radical form (DPPH-H). This reaction causes a visible decolorization from purple to yellow (an example is shown in Figure 4a), and the decrease in absorbance at 517 nm is used to quantify the free-radical scavenging ability of the films [61,62]. After incubation at 37 °C, DPPH activity decreased within 7 days, as shown in Figure 4b. This decrease over time may be attributed to the progressive release of curcuminoids from the fibroin matrix and to a partial degradation of the antioxidant compounds during incubation. However, the initial high antioxidant activity suggested an effective availability of curcuminoids during the early stages of wound healing, when oxidative stress is particularly relevant.

**Figure 5:**
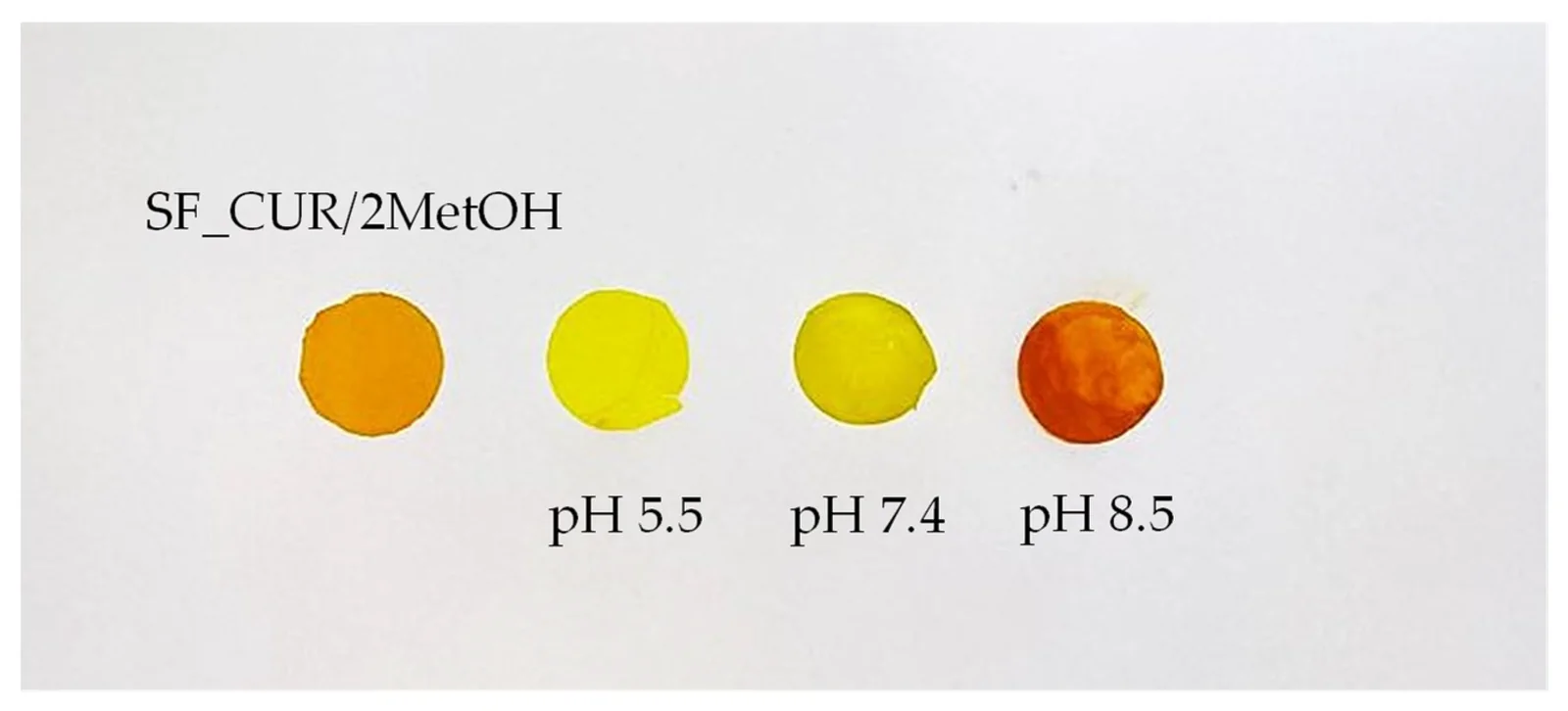
Visual assessment of the pH responsiveness of silk fibroin–curcuminoid films SF_CUR/2MetOH.

The pH responsiveness of the silk fibroin/curcuminoid films was evaluated through a visual colorimetric assessment and quantitatively evaluated using the CIE L* a* b* system (Table 1). As shown in Figure 5, the films displayed a clear color variation when exposed to buffers with different pH values. In particular, at neutral pH, the film exhibited L* = 81.30, a* = −10.97 and b* = 81.75. After exposure to acidic buffer, the film showed an increase in lightness (L* = 86.37) and a slight shift towards negative values of a* (−16.12) and positive values of b* (85.57), resulting in a yellow coloration. In contrast, after exposure to alkaline buffer, the film showed a decrease in lightness (L* = 52.90) and a marked increase in a* values (47.30) together with a decrease in b* values (60.97), resulting in a shift toward reddish/darker shades at higher pH values. This behavior can be attributed to the pH-dependent tautomeric equilibrium of curcuminoids, which modifies their electronic conjugation and consequently their visible light absorption. The overall color difference (ΔE) was calculated using the neutral condition as the reference, obtaining a value of 8.17 for the acidic condition and 68.08 for the alkaline condition. These results demonstrate a pH-dependent chromatic response of SF_CUR/2MetOH films, with the most pronounced color variation under alkaline conditions.

### 3.4. Biocompatibility Evaluation

#### 3.4.1. Cell Viability of 3T3 Fibroblasts Cultured in Contact with SF/2MetOH and SF_CUR/2MetOH Films

**Figure 6:**
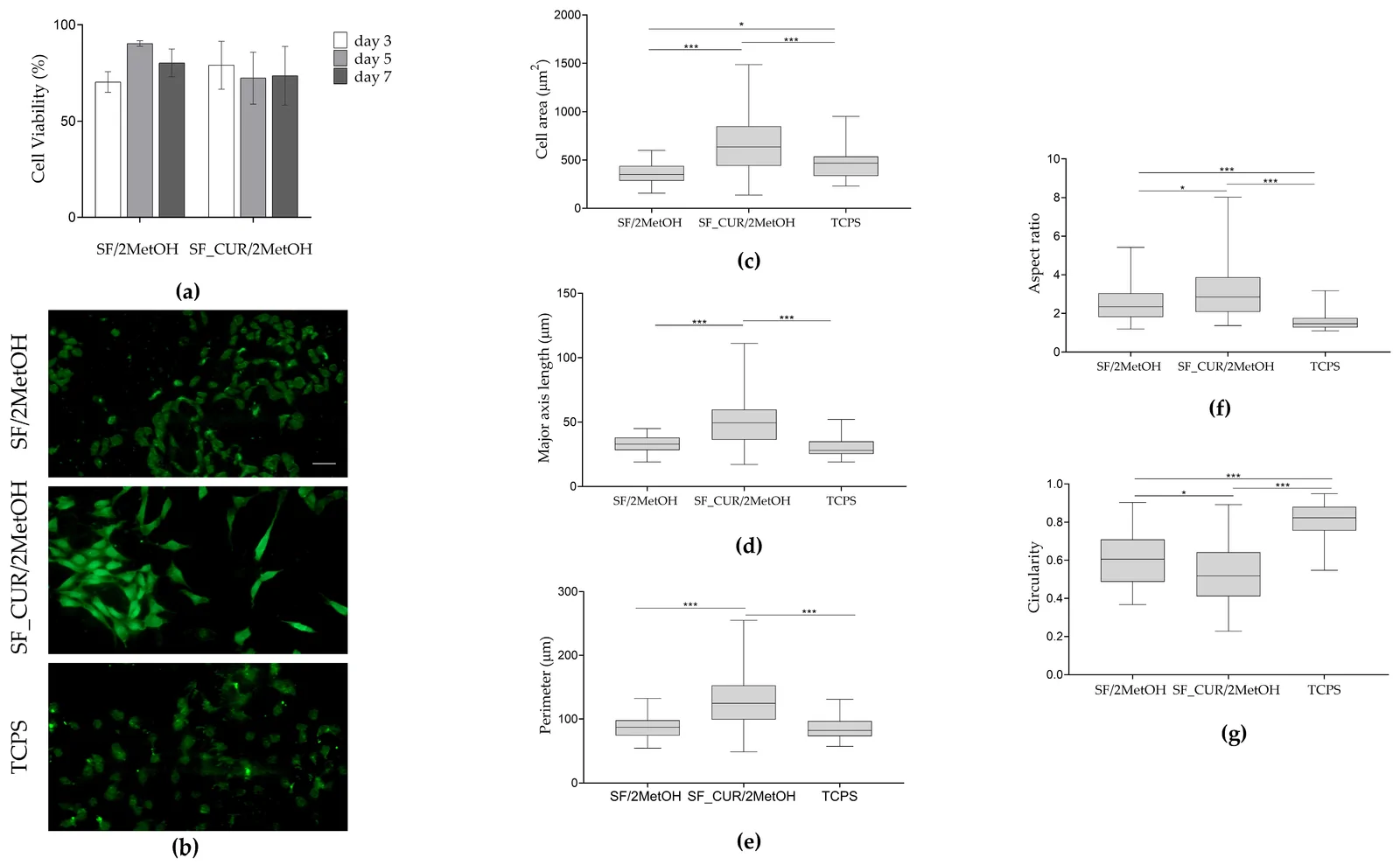
Effects of SF/2MetOH and SF_CUR/2MetOH films on cell viability. (**a**) Cell viability of 3T3 fibroblasts cultured in direct contact with the films was evaluated using MTT assay after 3, 5, and 7 days. Results were expressed as a percentage relative to the TCPS control. No significant differences in cell viability were observed among the experimental groups at any time point (*p* > 0.05). Although the SF_CUR/2MetOH group exhibited a slight decrease in viability over time compared with day 3, this trend was not statistically significant (*p* > 0.05). (**b**) Cell viability analysis of 3T3 fibroblasts using the Live/Dead assay. Images show 3T3 fibroblasts cultured in direct contact with the developed films after 7 days. Dead cells were not detected. Magnification at 20×; scale bar: 50 μm. (**c**) Cell area, (**d**) major axis length, (**e**) perimeter, (**f**) aspect ratio, and (**g**) circularity of 3T3 fibroblasts cultured in direct contact with the films and TCPS are compared (* *p* < 0.05; *** *p* < 0.0001).

The cytocompatibility of SF/2MetOH and SF_CUR/2MetOH films was evaluated by measuring the metabolic activity of 3T3 fibroblasts using the MTT assay. Cell viability was expressed as the percentage of absorbance relative to the TCPS control (Figure 6a). No statistically significant differences in cell viability were observed among the experimental groups at any time point (*p* > 0.05), indicating that the incorporation of curcuminoids did not significantly affect the viability of 3T3 fibroblasts compared with the SF/2MetOH film (Figure 6a).

The in vitro biocompatibility of the developed films was further evaluated in 3T3 fibroblast culture by the Live/Dead fluorescence staining at day 7. Cells cultured in contact with SF/2MetOH and SF_CUR/2MetOH films, as well as in TCPS control, exhibited a high percentage of viable cells, as evidenced by the predominant green fluorescence signal (Figure 6b). Conversely, negligible red fluorescence corresponding to dead cells was detected under both experimental conditions.

The morphometric analysis indicates that 3T3 fibroblasts cultured in contact with the SF-based films underwent morphological changes compared with the TCPS (Figure 6c–g). Specifically, fibroblasts cultured in contact with SF/2MetOH film exhibited a significant increase in cell area and aspect ratio (* *p* < 0.05 and *** *p* < 0.0001, respectively), together with a decrease in circularity (*** *p* < 0.0001), compared with the TCPS control condition (Figure 6c,f,g). These morphological changes were further enhanced by the addition of curcuminoids (Figure 6c–g). Indeed, fibroblasts cultured in contact with SF_CUR/2MetOH films display a greater cell area, major axis length, and perimeter compared with the other two experimental groups (*** *p* < 0.0001), a higher aspect ratio and a lower circularity compared with cells grown in contact with SF/2MetOH film and TCPS (* *p* < 0.05 and *** *p* < 0.0001, respectively).

Overall, these findings indicate that the incorporation of curcuminoids into silk fibroin-based films did not compromise their cytocompatibility, supporting 3T3 fibroblast viability and spreading and elongation over the culture period.

#### 3.4.2. Cytoskeleton Architecture of 3T3 Fibroblasts Cultured in Contact with SF/2MetOH and SF_CUR/2MetOH Films

**Figure 7:**
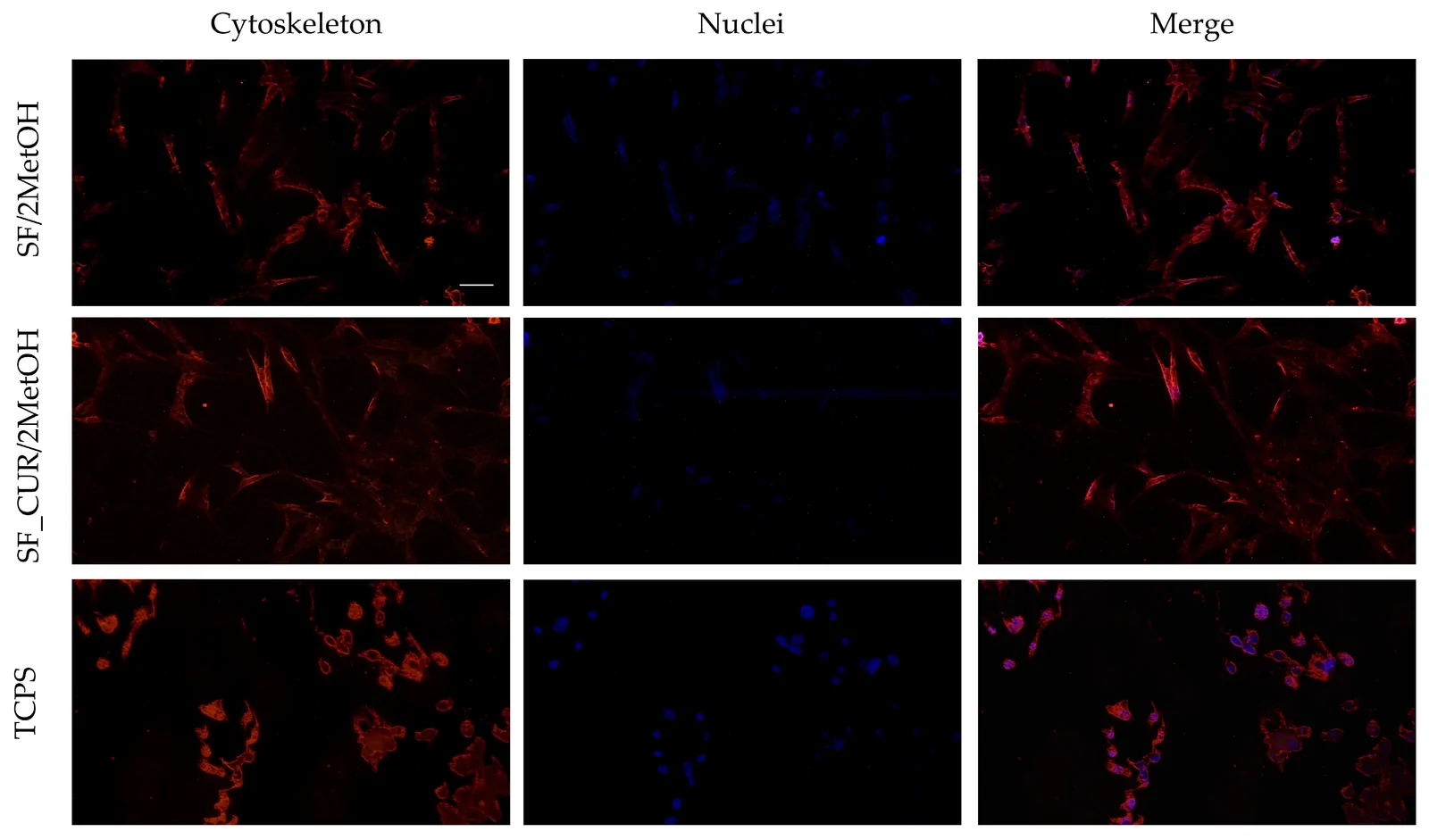
Cytoskeletal organization of 3T3 fibroblasts cultured in contact with SF/2MetOH and SF_CUR/2MetOH films after 7 days. No alterations in cytoskeletal morphology were observed in cells cultured on either SF/2MetOH or SF_CUR/2MetOH films, compared with the TCPS control. Images were acquired at 20× magnification; scale bar: 50 μm.

The organization of the 3T3 fibroblast cytoskeleton cultured in contact with SF/2MetOH and SF_CUR/2MetOH films was investigated by phalloidin-TRITC staining and compared with cells cultured on the TCPS control (Figure 7). Fluorescence microscopy analysis revealed a well-preserved cytoskeletal organization in all tested conditions. 3T3 fibroblasts exhibited a spread morphology with clearly defined and homogeneously distributed actin filaments. These findings indicate that the SF-based films, either alone or functionalized with curcuminoids (SF_CUR/2MetOH), provided a suitable microenvironment for maintaining cell adhesion and morphology, without inducing alterations in cytoskeletal organization after 7 days of culture (Figure 7). Cells cultured in contact with SF/2MetOH and SF_CUR/2MetOH films exhibited a more elongated and spread morphology compared with the TCPS control, suggesting enhanced cell-material interactions.

#### 3.4.3. Cell Migration in 3T3 Fibroblasts Grown in Contact with SF/2MetOH and SF_CUR/2MetOH Films

The wound-healing potential of the SF/2MetOH and SF_CUR/2MetOH films was evaluated through an in vitro scratch test using 3T3 fibroblast cultures. Cell migration and wound closure were observed immediately after scratch generation (T0) and after 2 and 3 days.

**Figure 8:**
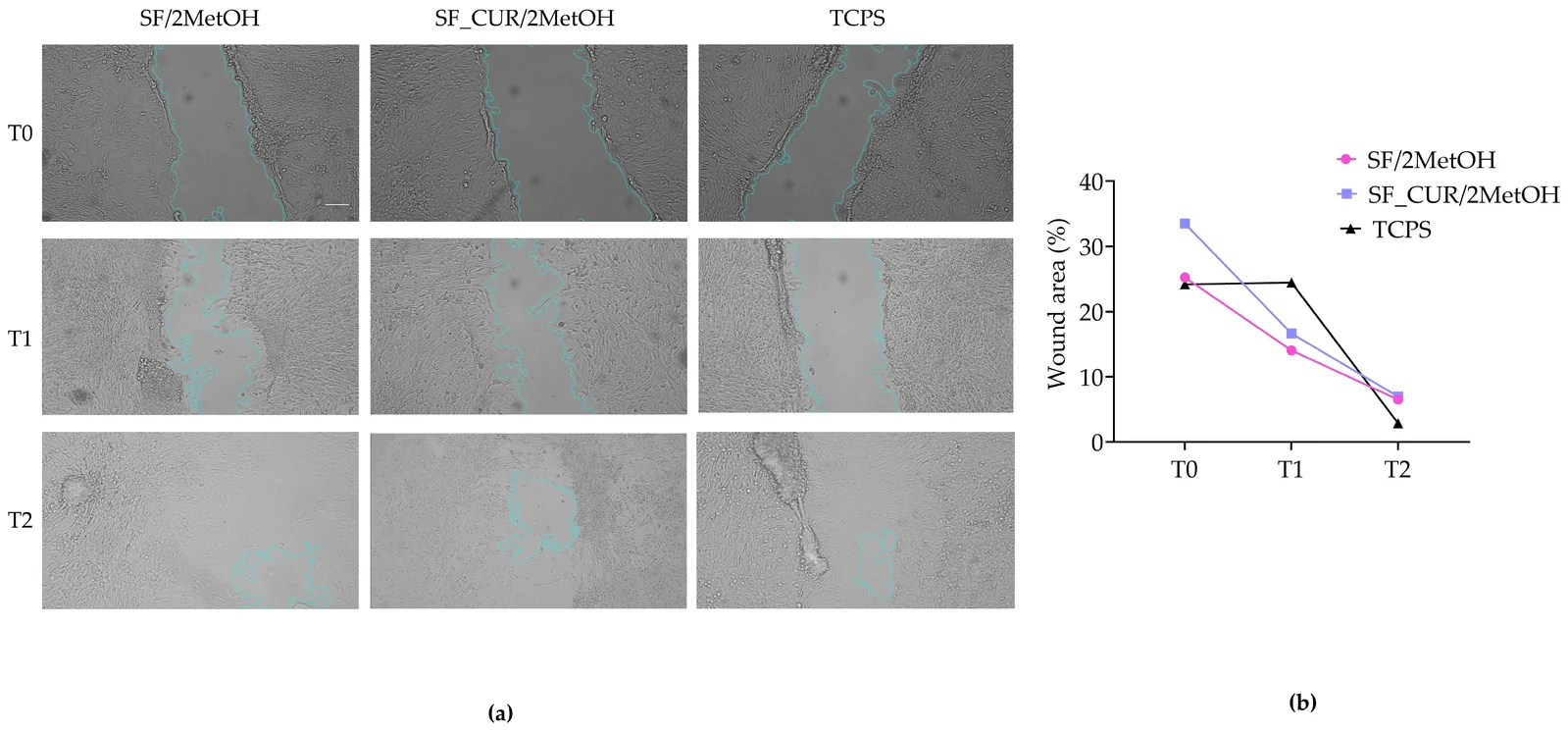
Wound healing analysis using an in vitro scratch assay. (**a**) Microscope images of wound closure of 3T3 fibroblasts grown in contact with SF/2MetOH and SF_CUR/2MetOH films and TCPS, the control. The analysis was performed after scratch generation (T0) and after 2 (T1) and 3 (T2) days. Magnification: 10×; scale bar 50 μm. (**b**) Quantification of wounded area invaded by 3T3 fibroblasts grown in contact with SF/2MetOH and SF_CUR/2MetOH films and TCPS, the control, up to 3 days.

Representative images and relative quantification obtained at each time point (Figure 8a,b) showed the progressive closure of the scratched area in 3T3 fibroblasts cultured in contact with the SF/2MetOH and SF_CUR/2MetOH films, as well as in TCPS (control group).

The analysis of the images revealed differences in the pattern of cellular repopulation among the experimental groups. Fibroblasts cultured in contact with the SF/2MetOH film showed a more homogeneous populated cell layer within the scratched region, compared with the other experimental groups. Fibroblasts cultured on the SF_CUR/2MetOH film displayed a more elongated and well-spread morphology, suggesting improved cell-material interactions (Figure 8a). Despite these morphological differences, wound closure proceeded efficiently. This aspect indicates that the incorporation of curcuminoids did not negatively affect 3T3 fibroblast migration up to 3 days.

Overall, both SF-based films supported 3T3 fibroblast migration and stimulated wound closure, demonstrating their appropriateness as substrates for tissue regeneration. These findings suggest that the SF_CUR/2MetOH film provides a favorable microenvironment for fibroblast migration, supporting its potential application as an advanced wound device for tissue regeneration.

## 4. Discussion

The present study aimed to develop a multifunctional silk fibroin biomimetic film incorporating naturally extracted curcuminoids to obtain an advanced wound dressing that combines the regenerative properties of silk fibroin with the antioxidant and pH responsiveness properties of curcuminoids.

Previous works have demonstrated that silk fibroin can be effectively used as a promising biomaterial for wound dressing applications, thanks to its biocompatibility, biodegradability and ability to be processed in the form of films, membranes, hydrogels and sponges [27,63]. In particular, from pre-clinical and clinical studies, it was demonstrated that silk fibroin films can reduce the time of wound healing and enhance the cutaneous regeneration [64,65]. The incorporation of curcuminoids in these films for wound dressing applications can improve their properties by also monitoring the stage of the wound repair process [37].

The first aspect of this work concerned the extraction of curcuminoids from *Curcuma longa*. The adopted methanolic extraction yielded a recovery efficiency of approximately 88% of the theoretical content of curcuminoids in rhizome powder. Although curcuminoids are frequently used in the literature in the form of pure commercial curcumin, the use of crude extracts represents a more sustainable and economically attractive approach, reducing purification steps while maintaining biological functionality [66].

The incorporation of curcuminoids into the silk fibroin matrix was achieved by dispersing the extracted powder in methanol, mixing it with the aqueous fibroin solution, and then obtaining film by air-drying and subsequent methanol-induced crystallization. The resulting films maintained excellent optical transparency despite the characteristic orange coloration imparted by curcuminoids, an important characteristic in wound dressing preparation for the visual monitoring of wound states [67,68].

The first part of the study was addressed to the investigation of the effects of curcuminoid incorporation into the fibroin matrix through chemical-physical analyses. Then, the best formulation for these innovative wound dressings was evaluated in its antioxidant, biocompatible and pH responsiveness behavior and wound healing properties.

The structural characterization performed by FT-IR analysis confirmed the successful stabilization of untreated silk fibroin (SF) through post-casting methanol crystallization with a shift in all characteristic amidic bands towards lower wavelengths for SF/2MetOH, indicating the transition from random coil Silk I structure to β-sheet-rich Silk II conformation, as extensively reported in the literature [24,69]. Conversely, the addition of a small amount of methanol into the precursor protein solution in SF/MetOH did not permit the structural transition into Silk II conformation. This is consistent with previous work where even a lower amount of methanol introduced into the fibroin solution provoked a decrease in β-sheet content in alcohol-treated films [70]. This transition is of fundamental importance to obtain a device that can adhere to the wound bed and absorb exudate and motivated why in this study the crystallized samples retain their integrity in an aqueous environment, whereas the non-crystallized samples completely dissolve after immersion in PBS [26,71]. This result is particularly relevant for wound dressing applications, since excessive swelling may lead to mechanical instability, whereas insufficient water absorption may limit the capacity of the material to maintain a moist environment [72,73,74]. For silk fibroin–curcuminoid films the presence of curcuminoids was masked in FT-IR spectra due to the overlap of the peaks of the two compounds in the same regions, together with intense absorbance of the protein signals and a relatively low curcuminoid-to-fibroin mass ratio but their incorporation in the films did not alter their ability to absorb fluids [60,62]. DSC analysis confirmed these structural changes with higher thermal stability and degradation temperatures of crystallized samples than untreated ones. The presence of curcuminoids further improved this stability, suggesting a possible stabilizing effect of curcuminoids, probably due to hydrophobic interactions between fibroin and curcuminoid domains, as previously reported in the literature [39].

The results obtained from degradation tests revealed a higher weight loss of silk fibroin films with respect to previous works [24]. This result is not inconsistent with FT-IR and DSC evidence, as the silk fibroin matrix behavior is also influenced by its microstructural organization and the distribution of crystalline and amorphous regions [75,76].

The direct addition of methanol alone and methanol/curcuminoids to the fibroin solution prior to film formation may have caused partial desolvation of the protein chains and a possible reorganization of the fibroin, which, following the crystallization treatment, resulted in a non-uniform distribution of β-sheet-rich regions. Indeed, a study conducted by Kasoju et al. demonstrated that methanol acts as a non-solvent in the fibroin-water system, inducing phase separation and changes in the structure of fibroin [77]. Similarly, the formation of β-sheets is a gradual process, dependent on the concentration of methanol, and is characterized by the presence of structural intermediate states [78]. From an applied perspective, this result could represent an advantage in the early stages of wound healing, as the higher degradation observed in the SF_CUR/2MetOH films could allow both the release of antioxidant curcuminoids and the gradual replacement of the biopolymeric matrix with newly formed tissue [79,80].

The incorporation of curcuminoids into silk fibroin films provided an additional biological functionality to the protein matrix, as they are characterized by strong antioxidant properties due to their phenolic hydroxyl groups and conjugated structure, which allow electron donation and stabilization of free radicals [81]. The release profile followed a first-order kinetic model, suggesting a concentration-dependent release behavior. Similar first-order release profiles have been previously reported for curcumin-loaded protein systems and also for other polymeric wound dressings and could be advantageous, as the high curcuminoids release in the first stages could reduce inflammation and oxidative stress [82,83]. The DPPH assay also confirmed that this incorporation significantly enhanced the radical scavenging ability of the fibroin-based materials, suggesting that these films could give antioxidant protection in the first stages of the wound healing process [83].

Moreover, the incorporation of curcuminoids determined a pH responsiveness of the SF_CUR/2MetOH films, a highly relevant feature as smart wound dressing. The films exhibited visible and quantitatively detectable color variations when exposed to buffers reproducing different wound healing conditions, demonstrating that curcuminoids retained their pH-sensitive optical properties after incorporation into the fibroin matrix. The observed color transition is associated with its acid base-dependent structural changes. Under acidic and near-neutral conditions, the molecule predominantly exists in its keto form, which is associated with the characteristic yellow appearance. Conversely, at alkaline pH, deprotonation processes promote the formation of enol/phenolate species, leading to an alteration of the conjugated electronic structure and consequently to a progressive shift toward orange-red coloration as pH increases [38,84]. This color change in the silk fibroin matrix due to curcuminoids could be advantageous, as chronic or infected wounds are usually associated with an alkali change in the wound microenvironment [10,11,12].

This pH color change, together with the transparency and the protein matrix of the obtained films, provides great advantages to the system: the silk fibroin matrix enhances regeneration properties, the transparency of the films permits the direct observation of the wound bed, while the presence of curcuminoids provides an optical signal that varies as a function of the wound microenvironment.

In the second part of the study, the cytocompatibility of SF/2MetOH and SF_CUR/2MetOH films using 3T3 fibroblasts has been verified as in vitro model.

Fibroblasts are key players in all three phases of wound healing. Indeed, they coordinate the repair process through the secretion of numerous molecules and dynamic interactions with other cell populations participating in the healing process [85].

The incorporation of curcuminoids into SFs did not compromise the in vitro cytocompatibility. Indeed, SF_CUR/2MetOH film maintained fibroblast metabolic activity and viability, comparable to those observed for SF/2MetOH film, indicating that curcuminoid functionalization did not induce cytotoxic effects. These results are consistent with the well-established biocompatibility of silk fibroin reported in previous studies, demonstrating that it stimulates fibroblast proliferation, keratinocyte migration, ECM deposition and re-epithelization and vascularization [64,86,87,88].

Cytoskeleton analysis further demonstrated that both SF-based films preserved normal cytoskeletal organization, with 3T3 fibroblasts displaying a well-spread morphology and organized actin stress fibers. The maintenance of cytoskeletal structure is recognized as an indicator of correct cell adhesion [89], suggesting that the physico-chemical features of both SF/2MetOH and SF_CUR/2MetOH films provide an appropriate cell microenvironment. The morphometric analysis also indicates that 3T3 fibroblasts cultured in contact with SF-based film exhibited a more spread and elongated phenotype, compared with the TCPS control condition. Interestingly, the incorporation of curcuminoids further enhanced these morphological variations, suggesting that the silk fibroin–curcuminoid film may induce greater fibroblast spreading and elongation, while enhancing cell–cell and cell-material interactions. These data are in agreement with previous findings reporting spread and elongated fibroblast morphologies after exposure to curcumin [90]. In addition, the wound-healing assay demonstrated that both SF/2MetOH and SF_CUR/2MetOH films supported cell migration, allowing progressive wound closure over the experimental period. In this context, these findings are particularly relevant, taking into consideration the crucial role of fibroblast recruitment and migration in the tissue repair process. Indeed, these cells contribute to ECM deposition and remodeling, supporting the repair of the damaged site [91]. Previous works have also reported that silk fibroin-based materials can regulate dermal fibroblast behavior, including cell morphology and ECM-related responses, promoting cellular processes involved in wound healing [92]. Thus, the migratory response observed in 3T3 fibroblasts exposed to both SF/2MetOH and SF_CUR/2MetOH films may contribute to their potential to support the wound-healing process. The enhanced elongation observed in 3T3 fibroblasts grown in contact with SF_CUR/2MetOH, together with their migratory behavior, may suggest a more favorable cell-material interaction due to the incorporation of curcuminoids. These data could be considered preliminary evidence of the potential of the silk fibroin–curcuminoid film to support relevant cellular processes involved in wound healing.

Collectively, these results suggest that silk fibroin acts as regenerative and structural support, ensuring cytocompatibility and fibroblasts adhesion and migration that are essential for tissue regeneration, while curcuminoids in the matrix act as bioactive component in response to the pH microenvironment, without altering the regenerative properties of silk fibroin.

## 5. Conclusions

In this study, silk fibroin-based films functionalized with curcuminoids were developed as a multifunctional platform to obtain an innovative smart dressing for wound treatment. In particular, the overall results demonstrated that the developed system (SF_CUR/2MetOH) combines support for tissue repair with the ability to provide visible information on the wound’s condition within a single biomimetic material, going beyond the traditional concept of dressings as passive wound protection.

The regenerative and structural component of the system was the silk fibroin matrix, capable of supporting the viability, proliferation and migration of fibroblasts, whilst the incorporation of naturally occurring curcuminoids conferred free radical scavenging activity and a visual optical response to pH changes, without altering the cytocompatibility of the protein-based matrix.

This multifunctional platform could provide a useful tool in the management of chronic or hard-to-heal wounds, where oxidative stress creates a pro-inflammatory environment within the wound bed that can impair healing. Therefore, by utilizing the intrinsic properties of curcuminoids as sensing molecules, it would be possible to monitor the progression of the wound’s healing status in real time, whilst at the same time the biomimetic fibroin matrix would ensure direct interaction with the cells involved in tissue regeneration processes.

However, further studies will be required to determine the translational potential of the system developed, including mechanical investigations, its validation under biologically relevant infected-wound conditions and deeper biological investigations into the anti-inflammatory and antimicrobial properties of curcuminoids.

Overall, the system developed, based on silk fibroin films and curcuminoids, represents a first step towards the development of a new-generation dressing capable of actively interacting with the wound environment.

## Figures and Tables

**Table 1:** L* a* b* values of SF_CUR/2MetOH films under acidic, neutral and alkaline conditions.

pH	L*	a*	b*	ΔE
5.5	86.37 ± 0.11	−16.12 ± 0.56	85.57 ± 0.03	8.17
7.4	81.3 ± 1.24	−10.97 ± 1.23	81.75 ± 0.88	/
8.5	52.90 ± 5.19	47.30 ± 5.87	60.97 ± 3.38	68.08

## Data Availability

Data are contained within the article.

## Abbreviations

The following abbreviations are used in this manuscript:

ECM	Extracellular matrix
PBS	Phosphate-buffered saline
ON	Overnight
RT	Room temperature
S.D.	Standard deviation
FT-IR	Fourier-transform infrared spectroscopy
ATR	Attenuated total reflectance
DSC	Differential scanning calorimetry
BCA	Bicinchoninic acid
DPPH	2,2-diphenyl-1-picrylhydrazyl
CIE	Commission International de l’Eclairage
ROIs	Regions of interest
TCPS	Tissue culture polystyrene
DMEM	Dulbecco’s modified Eagle medium
FBS	Fetal bovine serum
DMSO	Dimethyl sulfoxide
TRITC	Tetramethylrhodamine isothiocyanate
DAPI	4′,6-diamidino-2-phenylindole
